# Quality Control Standardization, Contaminant Detection and In Vitro Antioxidant Activity of *Prunus domestica* Linn. Fruit

**DOI:** 10.3390/plants11050706

**Published:** 2022-03-06

**Authors:** Mohd Amir, Ameeduzzafar Zafar, Rizwan Ahmad, Wasim Ahmad, Mohammad Sarafroz, Mohammad Khalid, Mohammed M. Ghoneim, Sultan Alshehri, Shadma Wahab, Sayeed Ahmad, Mohd Mujeeb

**Affiliations:** 1 Department of Natural Products and Alternative Medicine, College of Clinical Pharmacy, Imam Abdulrahman Bin Faisal University, Dammam 31441, Saudi Arabia; matahmad@iau.edu.sa (M.A.); rareiyadh@iau.edu.sa (R.A.); 2 Department of Pharmaceutics, College of Pharmacy, Jouf University, Sakaka 72341, Al-Jouf, Saudi Arabia; azafar@ju.edu.sa; 3 Department of Pharmacy, Mohammed Al-Mana College for Medical Sciences, Safaa, Dammam 34222, Saudi Arabia; wasimahmadansari@yahoo.com; 4 Department of Pharmaceutical Chemistry, College of Clinical Pharmacy, Imam Abdulrahman Bin Faisal University, Dammam 34212, Saudi Arabia; mskausar@iau.edu.sa; 5 Department of Pharmacognosy, College of Pharmacy, Prince Sattam Bin Abdulaziz University, P.O. Box 173, Al-Kharj 11942, Saudi Arabia; drkhalid8811@gmail.com; 6 Department of Pharmacy Practice, College of Pharmacy, AlMaarefa University, Ad Diriyah 13713, Saudi Arabia; mghoneim@mcst.edu.sa; 7 Department of Pharmaceutics, College of Pharmacy, King Saud University, Riyadh 11451, Saudi Arabia; salshehri1@ksu.edu.sa; 8 Department of Pharmacognosy, College of Pharmacy, King Khalid University, Abha 61421, Saudi Arabia; Shad.nnp@gmail.com; 9 Department of Pharmacognosy and Phytochemistry, School of Pharmaceutical Education and Research, Jamia Hamdard, New Delhi 110062, India; sahmad_jh@yahoo.co.in

**Keywords:** *Prunus domestica*, standardization, GC–MS, antioxidant, HPLC, microbial limits

## Abstract

The increase in the use of herbal medicines has led to the implementation of more stern regulations in terms of quality variation and standardization. As medicinal plants are prone to quality variation acquired due to differences in geographical origin, collection, storage, and processing, it is essential to ensure the quality, efficacy, and biological activity of medicinal plants. This study aims to standardize the widely used fruit, i.e., *Prunus domestica* Linn., using evaluation techniques (microscopic, macroscopic, and physicochemical analyses), advanced instrumental (HPLC, HPTLC, and GC–MS for phytochemical, aflatoxins, pesticides, and heavy metals), biological, and toxicological techniques (microbial load and antioxidant activities). The results revealed a 6–8 cm fruit with smooth surface, delicious odor, and acidic taste (macroscopy), thin-walled epidermis devoid of cuticle and any kind of excrescences with the existence of xylem and phloem (microscopy), LOD (15.46 ± 2.24%), moisture content (13.27 ± 1.75%), the high extractive value of 24.71 ± 4.94% in water:methanol (1:1; *v/v*) and with ash values in the allowed limits (physicochemical properties), and the presence of numerous phytochemical classes such as alkaloids, flavonoids, carbohydrates, glycosides, saponins, etc. (phytochemical screening). Furthermore, no heavy metals (Pb, Hg, Cd, Ar), pesticides, ad microbial limits were detected beyond the permissible limits specified, as determined with AAS, GC–MS analysis, and microbial tests. The HPTLC was developed to characterize a complete phytochemical behavior for the components present in *P. domestica* fruit extract. The parameters utilized with the method used and the results observed for the prunus herein may render this method an effective tool for quality evaluation, standardization, and quality control of *P. domestica* fruit in research, industries, and market available food products of prunus.

## 1. Introduction

The role of natural products in pharmaceutical biology is well established. Plants have been the primary source of medicine since ancient times. As per World Health Organization (WHO), >80% of the world’s population of traditional medicines and used for the treatment of disease. Many drugs nowadays are either biomimetics of naturally occurring molecules or have structures derived in whole or in part from natural patterns [1]. The promotion of alternative medicine use in developed countries has been hampered by the absence of evidence of documentation and strict quality control assessments. Data of all research conducted on traditional drugs must be kept and documented as well. With this particular issue, it is essential to ensure that the herbal crude drugs and their parts used as medicine are properly standardized. There are a variety of methods and techniques that can be used in process standardization, such as pharmacognostic, phytochemical, and contaminant analyses. It is possible to identify and standardize plant material using these methods and procedures. Characterization and proper quality assurance are important steps in ensuring the quality of herbal medicine, which aids in rationalizing its safety and effectiveness [2]. Active constituents and biomarkers can be determined with reasonable precision and reliability using the HPTLC method [3]. The WHO explained conventional medicines as containing various health practices, concepts, awareness, and theories integrating herbal, mineral, and/or animal-based remedies, spiritual medication, and physical exercises performed either individually or collectively to promote health or to cure, diagnose, or prevent diseases. Some terms related to natural medicines have been provided by the WHO, according to their definitions [4].

*Prunus domestica* Linn. fruit, usually known as aloo Bukhara, belongs to the *Rosaceae* family. It is a shrubby and small deciduous tree found in Europe, Egypt, India, and Pakistan’s high-altitude hilly regions of Kashmir and Swat [5]. Fresh, dried, or processed fruits from *P. domestica* have been known and consumed by humans since the ancient period. The fruit of *P. domestica* have carbohydrate, acid, and cellulose content similar to nectarines and peaches, and they all belong to the energy fruit foods [6]. Various antioxidants found in dried fruit have been shown to have anti-aging properties. Fever reducers are made from the bark of plants. Roots of *P. domestica* have astringent properties. Fatty oil from its seeds can be substituted for almond oil in many recipes [7]. *P. domestica* extract prevents fibroblast yield, enhances adrenal androgen production, and revives the secretory action of prostate and bulbourethral epithelium. There are various chemical ingredients found in *P. domestica*, i.e., flavonoids, flavonoid glycosides, abscisic acid, lignans carotenoid pigments, quinic acid, bipyrrole, and carbohydrates [8,9,10].

There are no previous reports available for the evaluation and standardization of *P. domestica* fruit. In the present research, the *P. domestica* fruit is subjected to various quality evaluation parameters ranging from microscopy to physicochemical, phytochemical, pharmacological, as well as toxicological analysis, in order to standardize the fruit as per the WHO guidelines. The quality evaluation may be very useful for herbal or p-pharmaceutical manufacturers and end consumers to utilize the established parameters for identification, quantification, selection, and usage of the best-quality prunus fruit.

## 2. Material and Methods

### 2.1. Reagents

Analytical grade chemicals and reagents were purchased from SD Fine Chemicals Ltd., Mumbai, India. Cadmium (Cd), lead (Pb), arsenic (As), and mercury (Hg) were obtained from Sigma Aldrich (St. Louis, MO, USA).

### 2.2. Collection and Authentication of the Drug Sample

The fruit of *P. domestica* was procured from the local market (New Delhi, India) and authenticated by a taxonomist from the Faculty of Science, Jamia Hamdard (New Delhi, India). The voucher specimen (Ref. PD/FP-369) was deposited into Herbarium and Museum, at Jamia Hamdard, New Delhi, India.

### 2.3. Macroscopic Evaluation

The macroscopical studies for the *P. domestica* were carried out visually through which the shape, color, taste, and odor were determined [11].

### 2.4. Microscopic Evaluation

For microscopic evaluation, a thin transverse section (TS) of the fruit was taken, stained with safranin (for lignification), and mounted to the microscopic slide. The sections were visualized and marked for the distinctive parts of the fruit by using a microscope. 

### 2.5. Physicochemical Evaluation

The physicochemical parameters of *domestica* fruit powder—namely, loss on drying, moisture content, total water-soluble content, acid insoluble content, and extractive value, were determined. The loss on drying was analyzed at 120 °C using a hot air oven (Thermofisher, Mumbai, India), and moisture content was analyzed by Karl Fisher’s titration method. The extractive value was determined by using various solvents—namely, chloroform, methanol, water: alcohol (1:1; *v/v*), and water, using the Soxhlet technique. The values were determined as per the WHO guidelines [4].

### 2.6. Phytochemical Tests (Chemical Classes Screening)

The extract of *P. domestica* fruit was subjected to different types of chemical tests to identify the presence of various natural compounds and chemical classes thereof. The phytochemical study was performed as per previously reported procedures [12].

### 2.7. Total Phenolics Content

Folin–Ciocalteu reagent method was employed for estimation of total phenolic content in *P. domestica* fruit [13]. Briefly, a dilute extract of the fruit (0.5 mL of 10 mg/mL) was mixed with Folin–Ciocalteu reagent (5 mL, 1:10 diluted with distilled water) and aqueous Na_2_CO_3_ (1 M, 4 mL). The mixture was kept for 15 min and total phenolic content was determined colorimetry at 765 nm (Shimadzu UV–Vis 1601, Tokyo, Japan) using gallic acid as standard. 

### 2.8. Total Flavonoid Content

The flavonoid content of the *P. domestica* fruit was estimated by the aluminum chloride colorimetric method [13]. Briefly, the methanolic diluted extract of the fruit (0.5 mL of 10 mg/mL) was mixed with 1.5 mL of methanol, 0.1 mL of 10% aluminum chloride, 0.1 mL of 1 M potassium acetate, and 2.8 mL of distilled water. The mixture was left standing for 30 min at 25 °C, and absorbance was measurement with a UV spectrophotometer at 415 nm (Shimadzu UV–Vis 1601, Tokyo, Japan). Rutin was used as a standard for flavonoid content determination.

### 2.9. Atomic Absorption Spectrometer (AAS) Study for Evaluation of Heavy Metals

An atomic absorption spectrometer was used for determination of heavy metals such as lead (Pb), cadmium (Cd), mercury (Hg), and arsenic (Ar) in fruit powder. The instrument condition used for AAS (Model # AA-240-FS); Cd (cadmium-EDL lamp, λ = 228.80 nm, fuel gas = acetylene at 2.5 L/min, support gas = air at 15.0 L/min), Pb (lead-EDL lamp, λ = 283.31 nm, fuel gas = acetylene at 2.5 L/min, support gas = air at 15.0 L/min), As (arsenic-EDL lamp, λ = 193.70 nm, fuel gas = argon at 5.5 L/min, support gas = air at 15.0 L/min), and Hg (mercury-EDL lamp, λ = 253.65 nm, fuel gas = argon at 5.5 L/min, support gas = air at 15.0 L/min). 

#### 2.9.1. Selection of Processing Parameters

The combination of fuel gas (acetylene) with subsidiary gas (air) in a mixture of 2.5: 15.0 L/min was used for the most effective separating of lead and cadmium. The mixture of 5.5:15.0 L/min of fuel gas (argon) with subsidiary gas (air) was employed as the most effective method for separating arsenic and mercury.

#### 2.9.2. Optimization of the Atomic Absorption Spectra

For sharp and sensitive signals, the positive ionization mode was used for atomic absorption spectrometry detection. A standard linear calibration curve (response vs. concentration) at three different concentrations, i.e., 0.50, 1.00, and 1.50 ppm was used for optimization. 

### 2.10. HPLC Determination of Aflatoxins Concentrations

#### 2.10.1. Sample Preparation

The developed methodology of association of analytical chemistry (AOAC) was used for the assessment of aflatoxins [14]. The acidic methanolic extract of *P. domestica* was neutralized with sodium chloride (NaCl) in n-hexane solution, followed by washing with dichloromethane solvent. The method was repeated 2 to 3 times to accumulate the dichloromethane layer, which was evaporated successively (2/3 mL). The resultant solvent was passed by silica gel column, followed by cleaning the column with the combination of benzene:acetic acid (9:1; *v/v*) and ether:hexane (3:1; *v/v*). The aflatoxins were eluted with 100 mL of dichloromethane:acetone (9:1; *v/v*), concentrated up to 5 mL, and desiccated with the help of inert nitrogen gas.

#### 2.10.2. Derivatization of Samples (Extract) and Standards

For the derivatization of the test samples, the dried extract was dissolved in a mixture of n-hexane (200 μL) and trifluoroacetic acid (50 μL), vortexed (30 s), and left standing for 5 min. Finally, a 1.95 mL solvent system (water:acetonitrile 9:1; *v/v*) was added to the above mixture for completion of the derivatization process. 

For derivatization of the standards, known concentrations (20, 40, and 80 ppb) of standard aflatoxins (B1, B2, G1, and G2) were taken and derivatized using the same aforementioned procedure for test sample derivatization.

#### 2.10.3. High-Performance Liquid Chromatography (HPLC–Fluorescence) Analysis

Waters Alliance e2695 separating module (Waters, Milford, MA, USA) with C-18 column (15 cm × 4.6 mm) was applied for determination of aflatoxins. Briefly, 20 μL of the derivatized samples (standards and test samples) were injected at 1 mL/min flow rate using water:acetonitrile:methanol (70:17:17; *v/v/v*) mobile phase. A fluorescent detector was used for recording the chromatogram. For quantitative analysis, peaks of the aflatoxins were compared with standard aflatoxins peaks (B_1_, G_1_, B_2_, and G_2_). 

### 2.11. GC–MS Analysis for Pesticides 

The AOAC official method (AOAC970.52/EPA525.5) was used for the determination of pesticides using GC–MS (Agilent 7890A GC system, Santa Clara, CA USA) [14]. GC–MS equipment was used (Agilent 7890A GC system, USA), and the AOAC970.52/EPA525.5 method was utilized for estimation of the pesticides. In detail, 50 mg of the sample was dissolved in methanol, followed by the addition of 50 mL of diethyl ether (added with 1 g of Na-oxalate) and petroleum ether. The mixture thus formed was vortexed for 1 min, and the organic layer was transferred into a separating funnel and added with 600 mL of water (saturated with NaCl). The aqueous layer was discarded, and this method was repeated 2 to 3 times. The resultant organic layer was mixed with sodium sulfate solution, collected, and evaporated (2–5 mL). This concentrated solution was mixed again with acetonitrile (30 mL) and petroleum ether (30 mL), passed through the column, and eluted with the help of diethyl ether. The solution obtained was concentrated (5 mL) using a rotavapor (Buchi, R-215, Flawil, Switzerland) and analyzed with the help of GC–MS.

### 2.12. HPTLC Finger Printing

For HPTLC analysis, CAMAG Linomat V (CAMAG) applicator with precoated silica gel F_254_ TLC-plates (Merck, Darmstadt, Germany) was used. The extracts of *P. domestica* fruit in different solvents (chloroform, methanol, water:alcohol (1:1; *v/v*) and water) were concentrated using a rotary evaporator and dried under reduced pressure using N_2_ gas inert condition. Finally, 5 mg/mL stock solution of each extract was prepared and spotted on TLC plates. For analysis, properly diluted stock solution was spotted via TLC plates, followed by the development of TLC plates in toluene:ethyl acetate:formic acid (5:4:0.5; *v/v/v*) solvent system for chloroform, methanol, aqueous:alcohol (1:1; *v/v*) extracts, whereas butanol:acetic acid:water (8:2:2; *v/v/v*) solvent system was used for aqueous extract TLC plate development. TLC plates were scanned by CAMAG scanner-3 at 366 nm, and photographs of the chromatograms were saved [15]. 

### 2.13. Microbial Contamination

The standard method as per the WHO guidelines was used for the determination of microbial load in the samples. The analysis included total fungal count, bacterial count (*Staphylococcus aureus, Salmonella ebony, Escherichia coli,* and *Pseudomonas aeruginosa*), as well other pathogen pathogens [16].

### 2.14. HPLC/DAD–DPPH Method for In Vitro Antioxidant Activity 

The HPLC (Shimadzu, Kyoto, Japan) instrument, consisting of a pump (LC-10 Ai, Kyoto, Japan), a system controller (SCL-10AVP), and a diode array detector (DAD-M10 AVP), was applied for analysis of in vitro antioxidant activity of all types of extract. RP-18 column (LiChrospher^®^, 250 mm × 4 mm, 5 µM) (Merck, Darmstadt, Germany) was used for chromatographic separation. Methanol/water (80:20, *v/v*) used as mobile phase at 1 mL/min flow rate in isocratic elution. For data acquisition and processing, LC10 software (Version 1.6) was used.

A procedure explained by Yen and Chen was used to estimate the antioxidant potential of the samples by measuring the radical-scavenging effect of 2, 2-diphenyl-1-picrylhydrazyl (DPPH) [17]. The DPPH (100 µM) was prepared in distilled water. Briefly, DPPH solution (1 mL) was added to fruit extract sample (1 mL) and standard (ascorbic acid) of different degrees of dilution (5–100 µg/mL) in each test tube with 3 mL of distilled water, vortexed vigorously, and kept at room temperature in dark light for 10 min. The samples were filtered via a nylon membrane filter (0.2 µM), and 20 µL aliquot samples were injected for HPLC analysis. For results interpretation, the decrease in the peak area of the samples was detected at 517 nm for 10 min of run time. The differences in the decline in peak area between the control and the samples were used for calculating the % inhibition. The following formula was applied to assess the % inhibition:

$$\%\;\mathrm{inhibition}=\frac{\left({\mathrm{PA}}_{C}-{\mathrm{PA}}_{S}\right)}{{\mathrm{PA}\;}_{C}}\times 100$$

PA_C_ = peak area of control, PA_S_ = peak area of samples.

## 3. Results and Discussion 

### 3.1. Macroscopical Observations

**Figure 1:**
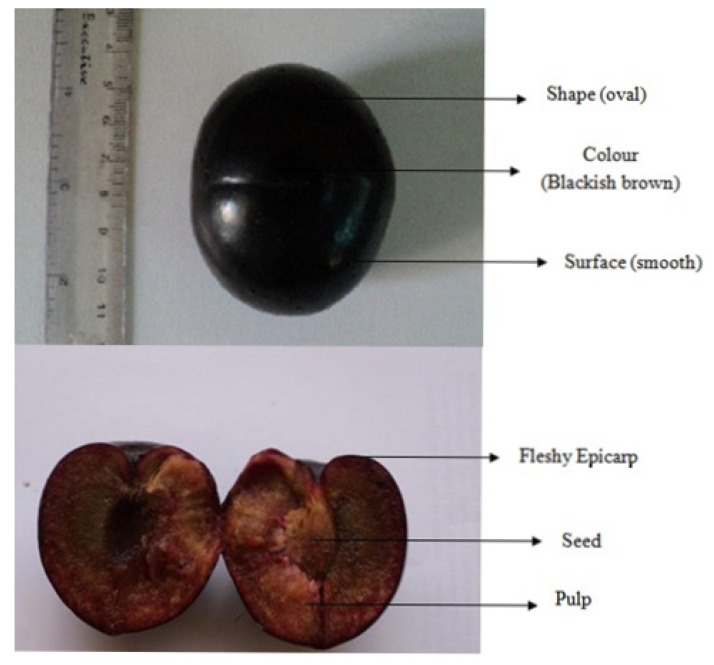
The macroscopy observation for *P. domestica* fruit sample.

Establishing the authenticity, identity, and purity of a medicinal plant can start with an organoleptic evaluation and should be tested prior to any in-depth assessment to verify the authenticity of the samples. The fruit’s color, shape, odor, and taste were found to be the most important characteristics for identifying the plant in a macroscopic study. The macroscopic observations for *P. domestica* revealed the fruit to have a blackish-brown color and an oval shape. The size of the fruit ranges from 6–8 cm in diameter and the surface is smooth (Figure 1). The odor of the fruit is delicious, and the taste is sweet to acidic.

### 3.2. Microscopic Observations

**Figure 2:**
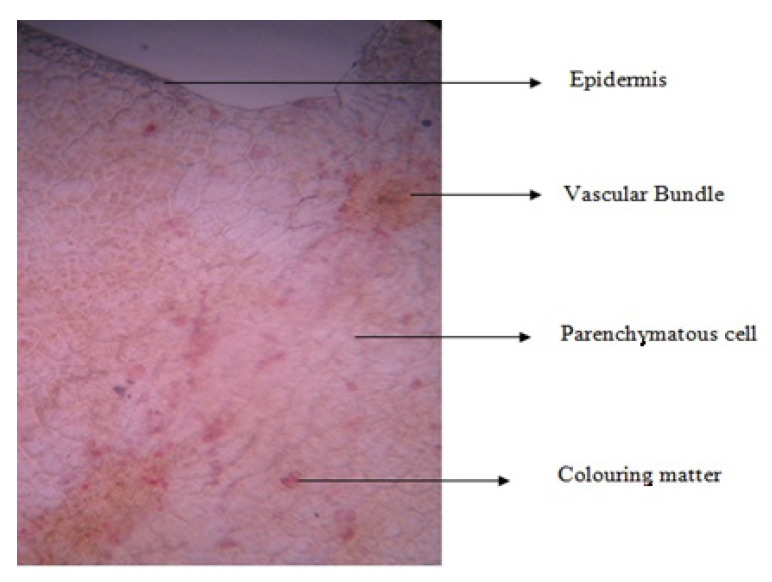
The microscopy observation for *P. domestica* fruit sample.

The transverse section of the fruit revealed an abundance of the rectangular-shaped parenchymatous cells in its ground tissue, thin-walled epidermis devoid of cuticle and any kind of excrescences, and uniformly distributed coloring matter possessing vascular bundles of xylem and phloem. The presence of crystals or any other orgastic content was not clear in the section (Figure 2). These specific characteristics are useful for the standardization of the fruit and may be used for the preparation of plant monographs in order to reduce the possibilities of adulteration.

### 3.3. Physiochemical Analysis

The results for various physiochemical tests are presented in Table 1. The assessment of the moisture content is an essential parameter for detecting inappropriate storage and handling of a sample. Herein, the moisture content of the drug as determined with the help of loss on drying (LOD) was revealed to be within the limits specified. The total ash is required for analysis of the purity of samples, i.e., the presence or absence of foreign inorganic matter (silica, metallic salts, etc.). It is a well-known fact that the total ash value may not be enough to determine the quality of a sample or herbal drugs, as the plant materials generally have major amounts of physiological ash (calcium oxalate and earthy matters in particular). Hence, acid insoluble ash value is more appropriate to determine the quality of herbal drugs in cases in which the evaluation for silica and earthy matter is desirable. The water-soluble ash consists of a water-soluble part of the total ash used to determine the amount of inorganic material observed in the herbal drugs [18,19,20]. A previous physiochemical study of this plant was compared and found to have very satisfactory results, but the previous study was on seeds of the plant, whereas the present study is on fruit [21].

The amount of an extract (extractive yield) in a particular solvent is mostly an approximation of the number of specific compounds that the drug contains. The drug should be extracted using a variety of solvents in order of their increasing polarity to obtain reliable and accurate values. Generally, petroleum ether, chloroform, methanol, water:alcohol (1:1; *v/v*), and water extractives are taken into account for finalizing the standards of a drug. The petroleum ether extract contains fixed oils, resins, and volatile materials. Although heating the extract (105 °C until constant weight) may evaporate the volatile oils, resins, coloring matters, and fixed oil still remain. Though alcohol solvents may dissolve almost all active compounds, they are usually used for analyzing the extractive index of the samples containing alkaloids, glycosides, resin, etc. Water is used for drug samples containing aqueous soluble materials as their major compounds. 

The results for physicochemical analysis (LOD, ash values, and extractive values) were found within the limits and comparable with pharmacopoeial standards. 

### 3.4. Phytochemical Analysis

Preliminary phytochemical screening (color reactions) of *P. domestica* fruit extract revealed numerous phytoconstituents classes, including alkaloids, terpenes, carbohydrate, phenolic compounds, flavonoids, and glycosides (Table 2).

### 3.5. Total Phenolic Content

In the plant kingdom, phenolics are the most common secondary metabolite. All of these different groups of constituents have received considerable attention as potential natural antioxidants because of their capability to act both as effective radical scavengers and metal chelators. Scientific evidence confirms that the antioxidant activity of phenol is attributed primarily to its redox properties, singlet oxygen quenchers, and hydrogen donor ability [22]. The total phenolic content in *P. domestica* extract was determined using the modified Folin–Ciocalteu method and found to be 1.98 ± 0.263% *w/w*.

### 3.6. Total Flavonoid Content

Similar to phenolics, flavonoids also possess significant antioxidant activity with considerable effects on human nourishment and health. Flavonoids work by either scavenging or chelating free radicals in the body [23]. The total flavonoid content in extract samples was assessed using the aluminum chloride colorimetric method, and the content was found to be 1.18 ± 0.484% *w/w* for *P. domestica* fruit.

### 3.7. Heavy Metals Determination via AAS

Due to their toxicity, persistence, and bioaccumulative nature, heavy metal contamination of traditional medicines and herbal samples is still a serious concern [24]. Heavy metals are well known for their side effects on several organs of the human body. For instance, continuing contact with lead may cause disturbance in the functioning of the nervous system, in addition to affecting kidney clearance [25]. The major sources of contamination with heavy metals such as cadmium, mercury, lead, and arsenic may be attributed to a contaminated environment during harvest or growth. The detection of contaminants (Pb, Cd, Hg, Ar) for *P. domestica* fruit sample in the linearity range of (0.5000–1.5000 ppm) revealed no resultant spectral peaks for any of the contaminants, as observed with AAS spectra (Table 3).

### 3.8. Aflatoxins Determination via HPLC

Aspergillus genus fungi produce aflatoxins, which are mycotoxins that can grow on a variety of food materials, spices, and herbal drugs. The main classification of aflatoxins includes B_1_, B_2_, G_1_, and G_2_; in this classification, B_1_ and G_1_ aflatoxins are considered more toxic than B_2_ and G_2_ because of the presence of extra double bond, which leads to the making of an electrophilic reactive epoxide in hepatic metabolism. Acute structural and functional injury to essential organs of the human body can occur because of this mechanism in action. It was considered that aflatoxin B1 can be a risk factor in the etiology of hepatocellular cancer in humans [26,27]. Environmental conditions such as temperature and high moisture during the cultivation and storage of herbal drugs support the growth and contamination of aflatoxins by the aspergillus genus. Herein, aflatoxins were analyzed by the HPLC method, and the results obtained revealed a lack of any such aflatoxins in the *P. domestica* fruit sample. 

### 3.9. Pesticides Determination via GC–MS

The ever-increasing demands for herbal drugs urge cultivation of the medicinal plants on a larger scale where the application of pesticides is also witnessed at an excessive level. Particular attention has been given to the impurity of organochlorine pesticides (OCPs) due to their toxicity and perseverance in the atmosphere and contamination by common pesticides [28,29]. The pesticides were evaluated by the GC–MS method, which revealed a pesticide-free sample of *P. domestica*. The pesticides analyzed in this study are listed in Table 4. 

### 3.10. HPTLC Finger Printing

**Figure 3:**
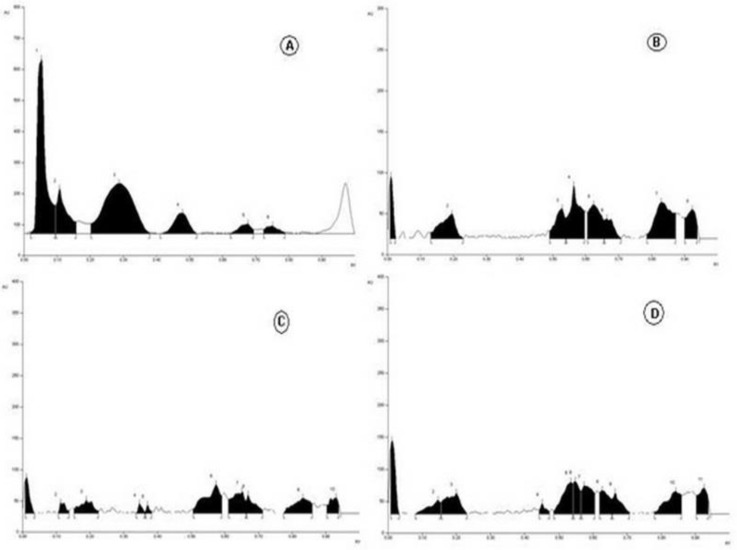
HPTLC fingerprint profile of chloroform (**A**), methanol (**B**), aqueous: alcohol (1:1 *v/v*) (**C**) and aqueous (**D**) of *P. domestica* fruit.

The technique of HPTLC is considered a mainstream approach to determine the quality of a sample with the help of fingerprint of plant standard chemicals. HPTLC has a high degree of sensitivity, which enables the detection of a variety of chemicals in a single run. The main objective of HPTLC estimation of *P. domestica* fruit was to develop an exceptional HPTLC chemical drugs pattern, representative of the whole chemical profile present in the fruit sample. A number of mobile phases were tried through the hit and trial technique for various solvent–extract ratios; acceptable separation of the compound was observed in the solvent system of toluene:ethyl acetate:formic acid (5:4:0.5; *v/v/v*), and butanol:acetic acid:water (8:2:2; *v/v/v*) for chloroform, methanol, aqueous:alcohol (1:1; *v/v*), and aqueous extract. The samples were applied, and a chromatogram was established in corresponding solvents and scanned at 366 nm. The results for solvent systems and peaks are presented in Table 5 and Figure 3.

### 3.11. Microbial Load for the Fruit Sample

**Figure 4:**
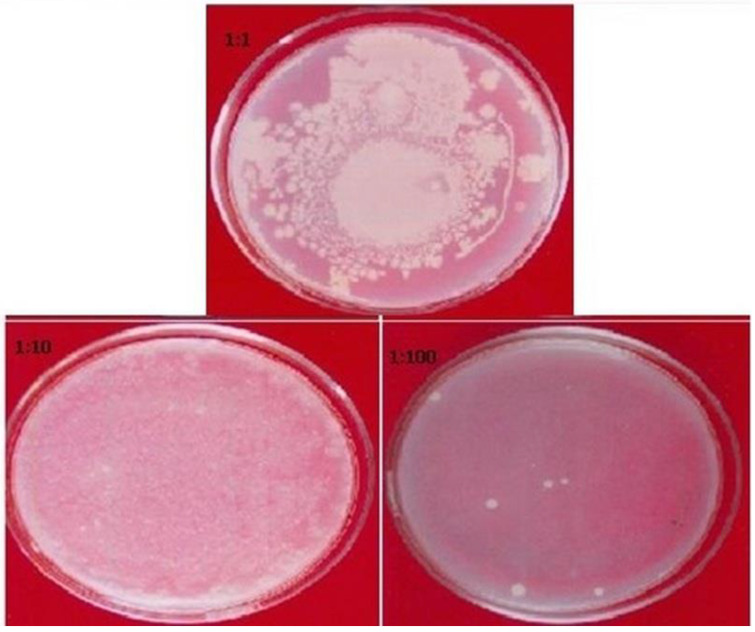
The plates representing the growth of microbe found in *P. domestica* fruit sample.

Generally, herbal drugs contain a variety of soil-borne microorganisms and molds. In order to ensure the safety of samples, the bioburden level is determined according to the procedure recommended by the WHO. The microbial profile for *P. domestica* was found within acceptable limits. The total microbial, mold, and yeast plate counts were 40 CFU/mL (≤10 CFU/mL as per the WHO guidelines). Furthermore, the pathogenic bacteria (*E. coli*, *Salmonella*, *Pseudomonas*, and *Staphylococcus*) were not found in the sample. The plates with microbe growth are shown in Figure 4.

### 3.12. HPLC/DAD–DPPH Method for In Vitro Antioxidant Activity 

**Figure 5:**
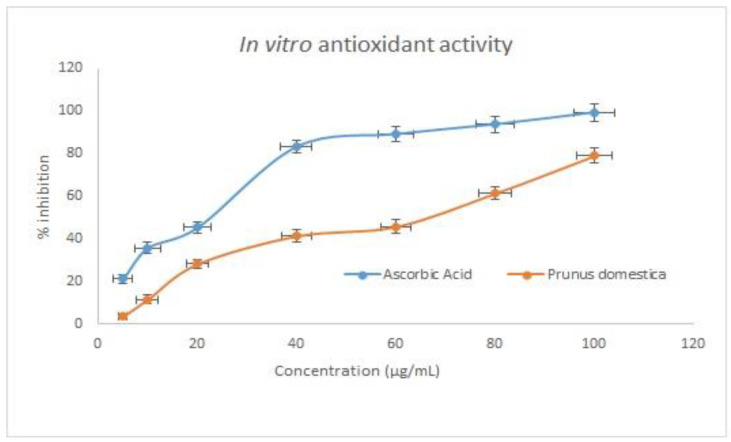
In vitro antioxidant activity.

Antioxidant activity in complex mixtures, such as herbal extracts, can be quickly assessed using HPLC–DPPH method [30]. The antioxidant potential for various extracts of the *P. domestica* fruit sample was determined by HPLC using a fluorescent detector. The half inhibition concentration (IC_50_) of *P. domestica* fruit extract was 34.28 ± 2.08 µg/mL, while the IC_50_ value for ascorbic acid was 16.30 ± 1.32 µg/mL (Figure 5). However, the results show that the developed method can be used to quickly screen for antioxidant compounds or more precisely radical scavenging activity of natural compounds. A previous antioxidant study of this plant was compared and found to have very satisfactory results, but the previous study was on seeds of the plant, whereas the present study is on fruit [31].

## 4. Conclusions

The results of physicochemical analysis, phytochemical screening, heavy metal detection, pesticides level, and in vitro antioxidant activity were useful in establishing the quality, safety, and efficacy of *P. domestica* fruit for its use as a potential drug candidate. The quality evaluation study herein may be a useful tool for the identification and standardization of the plant in terms of quality control of raw materials used in the nutraceutical or herbal formulation in industries. 

## Figures and Tables

**Table 1:** Summary of physicochemical parameters of *P. domestica* fruit (*n* = 5).

Parameters	% *w*/*w* (Mean ± SD)
LOD	15.46 ± 2.24%
Moisture content	13.27 ± 1.75%
Ash value	
Total ash	3.66 ± 0.257%
Acid insoluble ash	0.36 ± 0.082%
Water-soluble ash	2.83 ± 0.817%
**Successive extraction values**	
Petroleum ether	1.50 ± 0.13%
Chloroform	1.8 ± 0.35%
Methanol	15.21 ± 2.43%
Water:alcohol (1:1; *v/v*)	24.71 ± 4.94%
Water	20.80 ± 4.41%

**Table 2:** Phytochemical tests for detection of chemical classes in *P. domestica* fruit.

S. No.	Phytochemical Tests	Chloroform Extract	Alcoholic Extract	Aqueous Extract
1	Alkaloid	+	+	+
2	Sterols	+	+	+
3	Carbohydrate	−	+	+
4	Phenolic compound	+	+	+
5	Flavonoid	+	+	+
6	Amino acids	−	+	+
7	Saponin	−	+	+
8	Mucilage	−	−	−
9	Glycoside	−	+	+
10	Terpenes	+	+	−

+, present, −, absent.

**Table 3:** Heavy metal analysis of *P. domestica* fruit (*n* = 5).

	Mean ± SD (ppm)	Limit (Safe Up to) (ppm)
Lead	0.56301 ± 0.0089	10
Cadmium	0.00453 ± 0.0002	0.30
Mercury	0.441 ± 0.0246	0.50
Arsenic	1.182 ± 0.0203	3.0

**Table 4:** Different types of pesticides screened by AOAC method in selected fruit.

S. No.	Pesticide	Test Method
1	α-BHC	AOAC970.52/EPA525.5
2	β-BHC	AOAC970.52/EPA525.5
3	γ-BHC(Lindanee)	AOAC970.52/EPA525.5
4	δ-BHC	AOAC970.52/EPA525.5
5	Heptachlor	AOAC970.52/EPA525.5
6	Heptachlor_Epoxide	AOAC970.52/EPA525.5
7	α-Chlordane	AOAC970.52/EPA525.5
8	α-Endoulfan	AOAC970.52/EPA525.5
9	β-Chlordane	AOAC970.52/EPA525.5
10	Endrin	AOAC970.52/EPA525.5
11	Total DDE	AOAC970.52/EPA525.5
12	Total DDD	AOAC970.52/EPA525.5
13	Total DDT	AOAC970.52/EPA525.5
14	β-Endoulfan	AOAC970.52/EPA525.5
15	Endrin_Aldehyde	AOAC970.52/EPA525.5
16	Endoulfan_sulfate	AOAC970.52/EPA525.5
17	Aldrin	AOAC970.52/EPA525.5
18	Endrin_Ketone	AOAC970.52/EPA525.5
19	Methoxychlor	AOAC970.52/EPA525.5
20	Dieldrin	AOAC970.52/EPA525.5
21	Alachlor	AOAC970.52/EPA525.5
22	Butachlor	AOAC970.52/EPA525.5
23	Monochlorphos	AOAC970.52/EPA525.5
24	Phorate	AOAC970.52/EPA525.5
25	Mevinphos	AOAC970.52/EPA525.5
26	Dimethoate	AOAC970.52/EPA525.5
27	Malathion	AOAC970.52/EPA525.5
28	Methyl-parathion	AOAC970.52/EPA525.5
29	Chlorpyrifos	AOAC970.52/EPA525.5
30	Ethion	AOAC970.52/EPA525.5
31	Atrazine	AOAC970.52/EPA525.5
32	Simazine	AOAC970.52/EPA525.5
33	Diazinone	AOAC970.52/EPA525.5
34	Phosphamidon	AOAC970.52/EPA525.5
35	Fenitrothion	AOAC970.52/EPA525.5
36	Fenthion	AOAC970.52/EPA525.5
37	Phosalone	AOAC970.52/EPA525.5
38	Quinaphos	AOAC970.52/EPA525.5
40	Malaoxon	AOAC970.52/EPA525.5
41	Dichlorvos	AOAC970.52/EPA525.5
42	2,4-D	AOAC970.52/EPA525.5

**Table 5:** HPTLC fingerprint data of different extracts of *P. domestica* fruit.

S. No.	Sample	Solvent System	No. of Peaks and R_f_ Values
1	Chloroform extract	Toluene:Ethyl acetate:formic acid (5:4:0.5; *v/v/v*)	**(06)**; 0.05, 0.11, 0.29, 0.48, 0.68, 0.75
2	Methanolic extract	Toluene:Ethyl acetate:formic acid (5:4:0.5; *v/v/v*)	**(08)**; 0.01, 0.20, 0.53, 0.56, 0.62, 0.66, 0.83, 0.92
3	Aqueous:alcohol extract (1:1; *v/v*)	Toluene:Ethyl acetate:formic acid (5:4:0.5; *v/v/v*)	**(10)**; 0.01, 0.11, 0.19, 0.35, 0.37, 0.57, 0.65, 0.67, 0.83, 0.93
4	Aqueous extract	Butanol:acetic acid:water(8:2:2; *v/v/v*)	**(11)**; 0.01, 0.15, 0.20, 0.45, 0.54, 0.55, 0.57, 0.63, 0.67, 0.84, 0.93

## Data Availability

Not applicable.
